# Quality Improvement of Capsular Polysaccharide in *Streptococcus pneumoniae* by Purification Process Optimization

**DOI:** 10.3389/fbioe.2020.00039

**Published:** 2020-02-04

**Authors:** Chankyu Lee, Hee Jin Chun, Minchul Park, Rock Ki Kim, Yoon Hee Whang, Seuk Keun Choi, Yeong Ok Baik, Sung Soo Park, Inhwan Lee

**Affiliations:** ^1^R&D Center, EuBiologics Co., Ltd., Chuncheon-si, South Korea; ^2^Department of Biotechnology, Korea University, Seoul, South Korea

**Keywords:** pneumococcal capsular polysaccharide, purification, cetyltrimethylammonium bromide (CTAB) precipitation, cell wall polysaccharide, vaccine

## Abstract

*Streptococcus pneumoniae* is the causative agent of many diseases, most notably pneumonia. Most of the currently used vaccines to protect against this pathogen employ pneumococcal capsular polysaccharides (CPSs) as antigens, but purifying CPS of sufficient quality has been challenging. A purification process for CPS comprising conventional methods such as ultrafiltration, CTAB precipitation, and chromatography was previously established; however, this method resulted in high cell wall polysaccharide (CWPS) contamination, especially for serotype 5. Thus, a better purification method that yields CPS of a higher quality is needed for vaccine development. In this study, we significantly reduced CWPS contamination in serotype 5 CPS by improving the ultrafiltration and CTAB precipitation steps. Moreover, by applying an acid precipitation process to further remove other impurities, serotype 5 CPS was obtained with a lower impurity such as decreased nucleic acid contamination. This improved method was also successfully applied to 14 other serotypes (1, 3, 4, 6A, 6B, 7F, 9V, 11A, 14, 18C, 19A, 19F, 22F, and 23F). To assess the immunogenicity of the CPS from the 15 serotypes, two sets of 15-valent pneumococcal conjugate vaccines were prepared using the previous purification method and the improved method developed here; these vaccines were administered to a rabbit model. Enzyme-linked immunosorbent assay and opsonophagocytic assay demonstrated higher immunogenicity of the conjugate vaccine prepared using CPS produced by the improved purification process.

## Introduction

*Streptococcus pneumoniae*, which was first isolated by Louis Pasteur and George Sternberg independently in 1880 (Grabenstein and Klugman, 2012), is a gram-positive bacterium and major cause of pneumonia. The bacterium is also responsible for meningitis, otitis media, and other infectious diseases (Örtqvist, 1999; Hortal et al., 2000; Mitchell and Mitchell, 2010; Blasi et al., 2012). One of the most important virulence factors in *S. pneumoniae* is capsular polysaccharide (CPS), the types of which determine the serotypes of the pneumococcal bacterium (Dubos and Avery, 1931; Henrichsen, 1995). CPS composes the outer layer of *S. pneumoniae*, and more than 90 structurally different serotypes of *S. pneumoniae* have been reported thus far; 11 serotypes are known to cause over 70% of invasive pneumococcal diseases (Geno et al., 2015). Because pneumococcal CPS is a major factor of pathogenicity and involved in the antigen-specific immune response against *S. pneumoniae*, it has been a primary target for the development of pneumococcal disease vaccines by pharmaceutical companies worldwide (Melegaro et al., 2006).

In the cell wall of *S. pneumoniae*, cell wall polysaccharide (CWPS) coexists with CPS and is a common contaminant during the process of CPS purification (Xu et al., 2005). CWPS is a negatively charged molecule and structurally conserved, except for the number of phosphocholine groups, among the serotypes (Vialle et al., 2005; Brown et al., 2013). Because CWPS is known to cause an inflammatory response (Tuomanen et al., 1987) and antibodies against CWPS are known to be not protective despite its high immunogenicity (Nielsen et al., 1993), CWPS must be separated from CPS for pneumococcal vaccine development. However, it is difficult to remove CWPS from CPS because the molecules are covalently bound through peptidoglycan (Sørensen et al., 1990; Larson and Yother, 2017) except for serotypes 3 and 37. In this study, we developed an improved process for CPS purification from the fermentation broth of *S. pneumoniae* that reduces CWPS as well as other contaminants such as nucleic acids and proteins. We exploited the differences in molecular weight and electrostatic properties between CPS and CWPS and successfully improved the quality of isolated CPS. Higher-quality CPS and CPS with high CWPS content were then conjugated to the carrier protein CRM_197_ to overcome the limited effectiveness of polysaccharide vaccines since polysaccharide alone cannot elicit T-cell dependent immune responses (Beuvery et al., 1982; Lesinski and Westerink, 2001). Animal studies were conducted to compare the immunogenicity between the conjugate vaccines produced by the previous and new methods.

## Materials and Methods

### Cell Culture

To produce pneumococcal polysaccharides, each serotypes of *S. pneumoniae* were cultivated in Hemin free media in a 40L fermenter (Sartorius, BIOSTAT, D-DCU, Göttingen, Germany) at 37°C, pH 7.2. All 15 serotype of *S. pneumoniae* (1, 3, 4, 5, 6A, 7F, 9V, 11A, 14, 18C, 19A, 19F, 22 F, 23F) were obtained from Culture Collection University of Gothenburg (CCUG).

### Purification

#### Purification Before Process Optimization

After the cultivation of *S. pneumoniae*, 10% sodium deoxycholate (DOC) was added to the cell broth to achieve a final concentration of 0.1% to lyse the cells, followed by centrifugation at 7000 rpm. The supernatant was filtered using a 0.2 μm cut-off filter and subjected to ultrafiltration/diafiltration (UF/DF) against pure water at 10-fold volume of the supernatant by using a 50 kDa (for serotypes 4, 5, and 14) or 100 kDa (for serotypes 1, 2, 3, 6A, 6B, 7F, 9V, 11A, 18C, 19A, 19F, 22F, and 23A) cut-off cassette membrane filter (Hydrosart, Sartocon Ultrafiltration cassettes, Sartorius, Göttingen, Germany), depending on the size of the CPS. CTAB (10%) was added to achieve a final concentration of 0.7% to the diafiltered solution of CPS. For negatively charged CPS such as 1, 3, 4, 5, 6A, 6B, 9V, 11A, 18C, 19A, 19F, 22F, and 23A, CPS precipitated with some negatively charged impurities, including nucleic acids, and for neutral CPS such as 7F and 14, only impurities precipitated, leaving the CPS remain in the solution. Thus, after centrifugation of CTAB-treated solution, precipitates were collected for negative CPS, and supernatant was collected for neutral CPS. NaCl solution (1 M) was added to the precipitated CPS, and CPS in the supernatant was mixed with NaCl solution to achieve a final NaCl concentration of 1 M. Then, ethanol was added to CPS in 1 M NaCl solution (1st ethanol CPS precipitation) to a final concentration of 75% to precipitate CPS. Precipitates were recovered by centrifugation and dissolved in water. After treating 6% (w/v) sodium acetate and 0.5% (w/v) DOC, the pH of the solution was adjusted to 6.4 using 8 M acetic acid. Ethanol was added to this solution (ethanol impurity precipitation) to a final concentration of 20% (1, 3, 4, 5, 19A, and 19F) or 33% (6A, 6B, 7F, 9V, 11A, 14, 18C, 22F, and 23A) to precipitate the impurities. After centrifugation, the supernatant was collected and mixed with 6% (w/v) sodium acetate, and ethanol was added (2nd ethanol CPS precipitation) to achieve a final concentration of 75%. As in the 1st ethanol CPS precipitation process, in the 2nd ethanol CPS precipitation, CPS were precipitated, and these precipitates were collected by centrifugation and dissolved in water. Then, this CPS solution was sequentially diafiltered against Buffer I (50 mM Tris–HCl, 2 mM EDTA, 0.3% DOC), Buffer II (50 mM Tris–HCl, 2 mM EDTA), and Buffer III (150 mM NaCl). Afterward, buffer exchange to water was performed by diafiltration for hydroxyapatite (HA) chromatography, during which impurities will bind to the column. For the HA chromatography, column was equilibrated with PBS (10 mM phosphate buffer, 150 mM sodium chloride, pH 7.4) before sample loading. The flow through (F/T) was recovered for the final UF/DF and formulation.

#### Improved Purification Method

**FIGURE 1:**
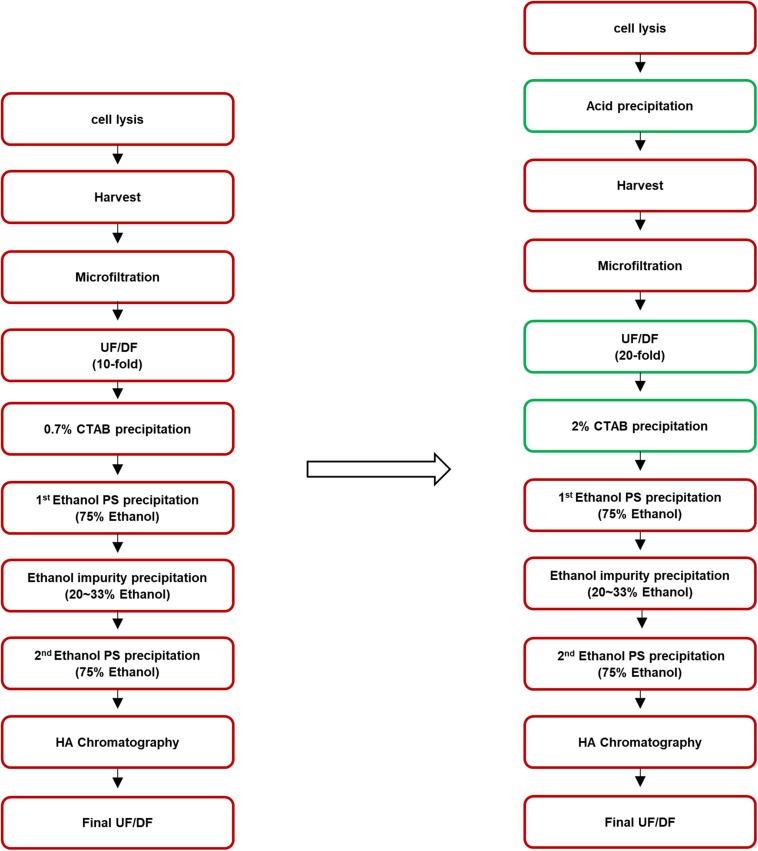
Process flowchart of pneumococcal polysaccharide purification. The method before improvement is presented in the left and the improved steps are indicated in the green box in the right.

To improve the process, after cell lysis, acid precipitation was performed, during which the pH of the cell broth was adjusted to 5.0 by adding acetic acid to remove impurities such as soluble proteins. After the acid precipitation, extensive UF/DF against pure water at 20-fold volume of the supernatant, which was twice the volume used in the former method, was performed to remove low molecular weight molecules including CWPS. CTAB concentration was also increased, from 0.7 to 2%. The purification steps before and after optimization are presented in Figure 1.

### Gel Permeation Chromatography (GPC)

To determine the molecular sizes of pneumococcal CPS from each serotype, gel permeation chromatography (GPC) was performed using the Waters e2695 system equipped with the TSKgel G6000PW/5000PWXL column (2× bed column). Dextran standard (American Polymer Standard Corporation) was used to calculate the molecular weight of CPS, as dextran and pneumococcal CPS have similar characteristics.

### Nuclear Magnetic Resonance (NMR) Spectroscopy

Concentrations of CWPS were measured by 1D ^1^H-NMR spectroscopy. CPS samples were dissolved in 10% D_2_O solution, and the 1D spectra were recorded with a Bruker Avance Neo 600 (600 MHz) spectrometer. NMR data were processed, and the spectra were analyzed in Topspin^®^ software. CWPS content was calculated as the relative peak area ratio of CWPS to CPS.

### Measurement of Proteins and Nucleic Acids

Proteins and nucleic acids as contaminants in CPS were measured by a Lowry protein assay kit (Thermo Fisher Scientific, Waltham, MA, United States) and NanoDrop spectrophotometer.

### Activation of PnPSs

Purified CPS larger than 700 kDa was fragmented by a Microfluidizer^®^ (Microfluidics, United States) at 30.000 psi to reduce the size. An ADH linker molecule was covalently bound to 5 mg/mL of fragmented CPS between 250 and 700 kDa in size using 1-cyano-4-diethylaminopyridinium (CDAP) and tri-ethylene amine (TEA) as described in literatures (Lees et al., 1996; Suárez et al., 2008). Briefly, CDAP in acetonitrile was added to 5 mg/mL of the polysaccharide solution at 1/10 of the volume (50 mg/mL), and after 1 min, 0.21 M of TEA was added at the same volume of CDAP (50 mg/mL, in acetonitrile). After 3 min, the same volume of 0.5 M ADH with 50 mg/mL CDAP (in acetonitrile) was added to the solution, and the activation reaction was performed for 15 h at 4–10°C with shaking at 200–300 rpm using a magnetic stirrer. Activated polysaccharides were confirmed by measuring the degree of activation, which shows the concentration of ADH molecules bound on the polysaccharide chains, by TNBS (2,4,6-trinitrobenzene sulfonic acid) assay (Snyder and Sobocinski, 1975).

### Conjugation of Activated PnPSs to CRM_197_

Activated PnPSs were conjugated to CRM_197_ using 1-ethyl-3-(-3dimethylaminopropyl) carbodiimide hydrochloride (EDAC) as a catalyst in 0.1 M MES buffer (pH 6.0). After the conjugation reaction proceeded at 4°C, EDAC and unbound CRM_197_ were removed by DF with a 100-kDa cut-off membrane filter, and Polysorbate 80 was added to a final concentration of 0.005%.

### Formulation of 15-Valent Pneumococcal Conjugate Vaccines

All 15 CPS-CRM_197_ conjugates were adsorbed on aluminum phosphate adjuvant. Each vial of vaccine (0.5 mL) contained 125 μg of aluminum phosphate and 2.2 μg of CPS (except 6B, which contained 4.4 μg).

### Animal Study

All animal studies were approved by the Institutional Animal Care and Use Committee (approval No.: 17B025) according to Animal Protection Law (13023, January 20th, 2015). To test the immunogenicity of the 15-valent pneumococcal conjugate vaccines, 0.5 mL of Prevnar 13^®^ and formulated vaccine were intramuscularly injected into New Zealand white rabbits weighing 1.16–1.30 kg. After the first injection, two boosting injections were conducted at 2-week intervals, and serum samples were collected 6 weeks after the first injection.

### Enzyme-Linked Immunosorbent Assay (ELISA)

Enzyme-linked immunosorbent assay units for 15 serotypes were measured by a modified method described by Concepcion and Frasch (2001). Microtiter plates were coated with 1 μg/mL of serotype-specific pneumococcal CPS antigens and incubated for 5 h at 37°C. Rabbit sera were pre-absorbed with pneumococcal C-polysaccharide (Statens Serum Institut) and each serotype of CPSs (American Type Culture Collection) for 30 min at room temperature before serial dilution. Serially diluted serum was added onto a washed plate, and the plates were incubated at room temperature for 1 h. After 1 h of incubation, the plates were washed, and HRP-conjugated goat anti-rabbit IgG (AB Frontier) was added. Following a 1-h incubation and washing, substrate (3,3′,5,5′-tetramethylbenzidine, TMB) was added to all wells. The optical density of each well was measured at 450 nm using an ELISA plate reader. *t*-test was used to evaluate the increase of the serotype specific IgG levels in the sera. *p* values of <0.05 was considered as statistically significant and all statistical analysis was performed by GraphPad Prism v5.01 (GraphPad, La Jolla, CA, United States).

### Opsonophagocytic Activity Assay (OPA)

The functional activity of antibodies in sera was measured by the standard opsonophagocytic activity assay (OPA) (Burton and Nahm, 2006). HL-60 cells (Korean Cell Line Bank, KCLB No. 10240) were used as effector cells, which express complement receptors CR1 and CR3 (for iC3b and C3b). HL-60 cells were maintained, passaged, and differentiated into granulocytes with dimethylformamide. HL-60 cells were allowed to phagocytose bacteria in the presence of sera containing anticapsular antibodies and baby rabbit complement (Pel-Freez Biologicals). The assay was performed on microtiter plates, and duplicate samples from each well were plated on THYE agar plates. The results were expressed as an opsonic index, which is the reciprocal of the serum dilution at 50% killing as compared to bacterial growth in controls without serum.

## Results

### Molecular Weights of CPS and CWPS

**FIGURE 2:**
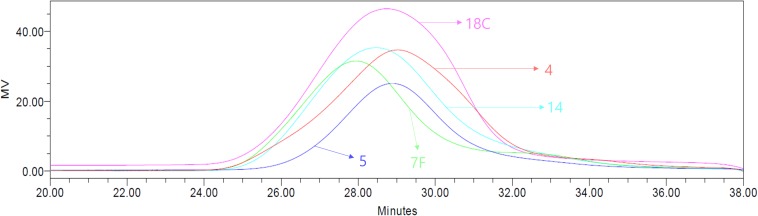
Molecular weights of serotypes 4, 5, 7F, 14, 18C measured by GPC. The serotypes of 4, 5, 7F, 14, and 18C are shown in red, blue, green, cyan, and pink colors, respectively.

The molecular weights of CWPS were measured by GPC to determine if CWPS can be separated from CPS based on size differences (Figure 2). As summarized in Table 1, the molecular weights of CPS were 951.0, 1002.1, 1275.1, 1153.8, and 1128.3 kDa for serotypes 4, 5, 7F, 14, and 18C, respectively. CWPS has a molecular weight of approximately 10 kDa, indicating that the large difference in size can be utilized for separation.

**TABLE 1:** Calculated molecular weights of serotypes 4, 5, 7F, 14, and 18C measured by GPC.

Serotype	4	5	7F	14	18C
Molecular weight (kDa)	951.0	1002.1	1275.1	1153.8	1128.3

### Concentrations of CWPS, Nucleic Acids, and Protein Impurities in Serotype 5 CPS

**FIGURE 3:**
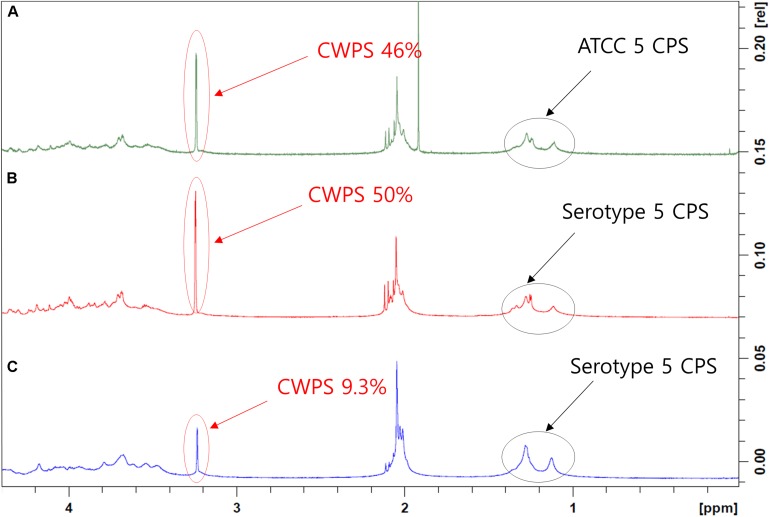
1D ^1^H-NMR spectra of pneumococcal polysaccharide of serotype 5. **(A)** Pneumococcal polysaccharide of ATCC standard. **(B)** Pneumococcal polysaccharide purified using the method before improvement. **(C)** Pneumococcal polysaccharide purified using improved method. The peaks from CPSs and cell wall polysaccharides are indicated by arrows. The peaks in the 1–1.5 ppm region are three methyl groups in type 5 CPS, and the peak that appeared at 3–3.5 ppm is phosphocholine groups in CWPS.

**FIGURE 4:**
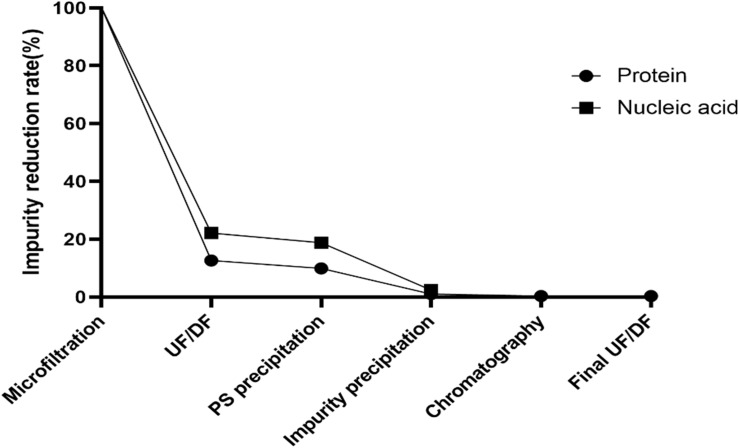
Reduction rates of impurities in each steps of improved method. Reductions of protein and nucleic acid contaminants are indicated by circle and square dots, respectively. Microfiltration is a filtering process using 0.2 μm cut-off capsule filter while UF/DF process is a process to remove low molecular contaminants using 50 kDa cut-off filter. PS and impurity precipitation were carried out by using CTAB and ethanol, respectively. Hydroxyapatite (HA) column was used in chromatography process and buffer exchange to the final formulation was performed in final UF/DF process.

Cell wall polysaccharide content in serotype 5, determined to be 50% by NMR, was the highest among all 15 serotypes (data not shown) and similar to that of a comparator CPS (ATCC standard pneumococcal serotype 5 polysaccharide) (Figures 3A,B). After optimization of the purification processes, which included changes in the conditions of CTAB addition and ultrafiltration, the CWPS concentration fell to 9.3% (Figure 3C). Despite the decrease in CPS yield, the levels of other contaminants such as nucleic acids were also reduced, as was the concentration of CWPS, after applying the improved processes (Table 2). There was a small increase in protein contamination after process optimization; however, it is thought to be within a batch variation range. The reduction rates of nucleic acids and proteins in CPS from serotype 5 were monitored step-by-step in each process, revealing that the majority of the impurities were removed by the intensive microfiltration step (Figure 4). During UF/DF, proteins and nucleic acids were reduced by 85.7 and 75.6%, respectively. When impurity precipitation was performed using ethanol, proteins and nucleic acids decreased by 98.6 and 96.7%, while in the chromatography step, levels decreased by 99.3 and 99.6%. Therefore, changes in UF/DF and impurity precipitation processes resulted in the most dramatic decreases in nucleic acid contaminants from serotype 5.

**TABLE 2:** Comparison of the yields and content of impurities of serotype 5 before and after process optimization.

	Before improved	After improved
CPS yield (g/L)	0.26 ± 0.05	0.17 ± 0.07
Proteins (%)	3.16 ± 2.08	4.34 ± 1.19
Nucleic acids (%)	5.13 ± 1.78	0.03 ± 0.01
CWPS (%)	≥50	9.3

### Concentrations of CWPS, Nucleic Acids, and Protein Impurities in 14 Other Serotypes

**FIGURE 5:**
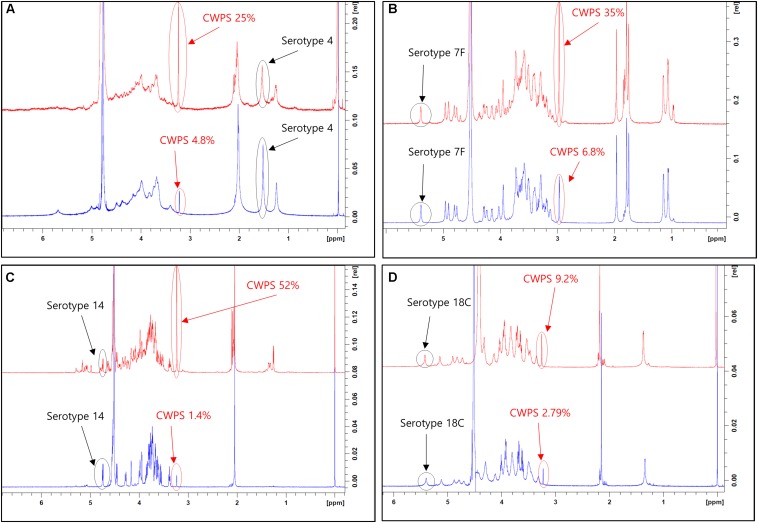
1D ^1^H-NMR spectra of pneumococcal polysaccharide of serotypes **(A)** 4, **(B)** 7F, **(C)** 14, **(D)** 18C. Peaks from capsular polysaccharides and cell wall polysaccharides are indicated by arrows in each panel. The peaks from pyruvyl ketal (type 4) and anomeric protons (types 7F, 14, and 18C) are chosen and identified in the spectra since these chemical groups are unique and easily identifiable from the peaks from phosphocholine groups in CWPS.

**FIGURE 6:**
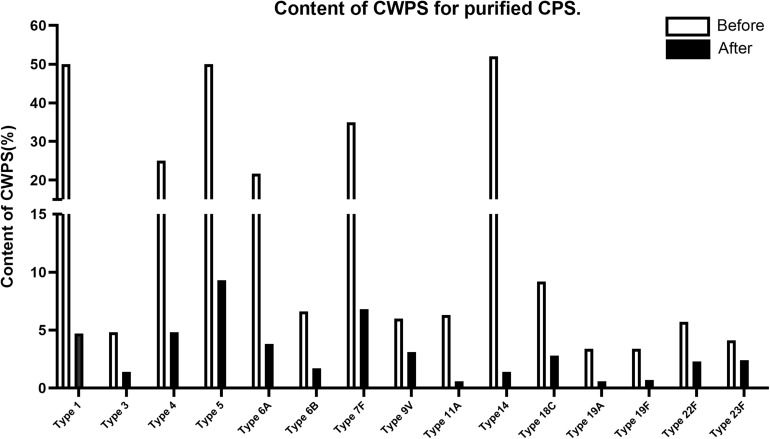
CWPS contents in each of 15 pneumococcal polysaccharides (1, 3, 4, 5, 6A, 6B, 7F, 9V, 11A, 14, 18C, 19A, 19F, 22F, 23F). CWPS contents are presented as a percentage of the ratios of cell wall polysaccharides to capsular polysaccharides. The comparison of before and after the process improvement are presented in the graph for each serotype.

This improved purification method was then applied to four other serotypes (4, 7F, 14, and 18C) that also exhibited high CWPS concentrations used the previous method. The NMR spectra of CPS produced by each method were compared (Figure 5). CWPS concentration was decreased from 25 to 4.8% for serotype 4 (Figure 5A), 35–6.8% for 7F (Figure 5B), 52–1.4% for 14 (Figure 5C), and 9.2–2.8% for 18C (Figure 5D). The production yield of CPS and concentrations of nucleic acids and proteins from serotypes 4, 7F, 14, and 18C were not affected by modification of the processes (Table 3), indicating that CWPS concentrations were efficiently reduced by the improved method, without affecting other factors. When the improved method was applied to other serotypes (1, 3, 6A, 6B, 9V, 11A, 19A, 19F, 22F, and 23F), decreases in CWPS were consistently observed, indicating that the method is generally applicable (Figure 6). In particular, notable decreases were observed in serotypes 1, 4, 5, 6A, 7F, and 14.

**TABLE 3:** Comparison of the yields and content of impurities of serotypes 4, 7F, 14, and 18C.

	CPS yield (g/L)		Proteins (%)		Nucleic acids (%)	
	Before	After	Before	After	Before	After
Serotype 4	0.26 ± 0.01	0.24 ± 0.02	0.95 ± 0.15	0.86 ± 0.03	0.19 ± 0.06	0.20 ± 0.02
Serotype 7F	1.59 ± 0.18	1.36 ± 0.01	0.94 ± 0.18	0.69 ± 0.07	0.09 ± 0.01	0.04 ± 0.01
Serotype 14	0.19 ± 0.02	0.10 ± 0.02	3.47 ± 0.34	2.73 ± 0.57	0.12 ± 0.05	0.11 ± 0.05
Serotype 18C	0.88 ± 0.06	1.17 ± 0.04	0.03 ± 0.01	0.01 ± 0.01	0.07 ± 0.05	0.04 ± 0.03

### Impact of the Increased Quality of CPS on Immunogenicity

**FIGURE 7:**
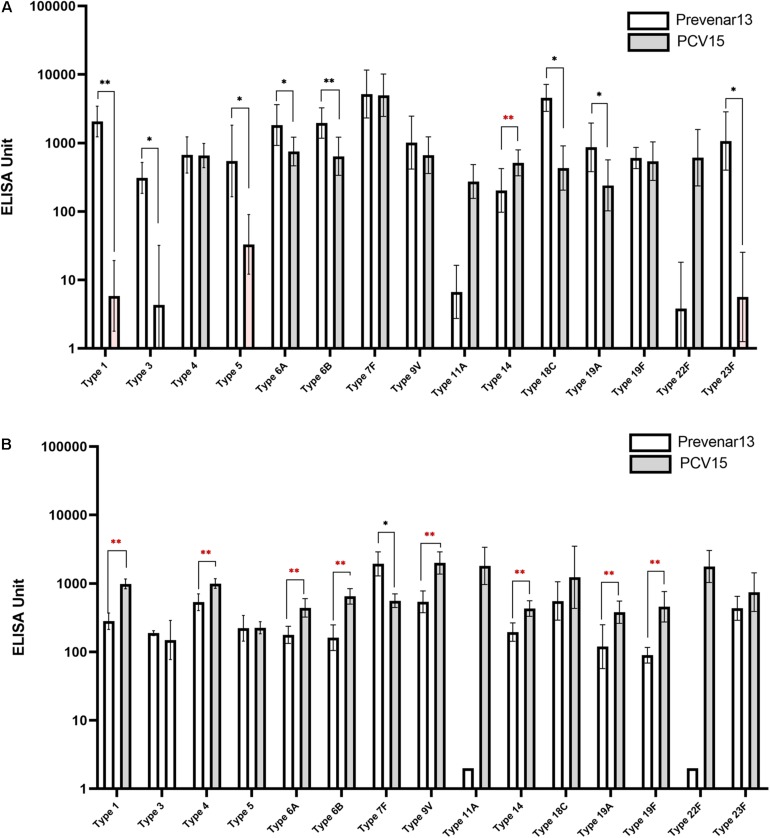
Immune responses against pneumococcal polysaccharides in mice measured by ELISA. **(A)** Before the process optimization. **(B)** After the process optimization. Prevenar 13^®^ was used as a comparator vaccine and PCV15 indicates the polysaccharide-carrier protein conjugate produced in this study by Eubiologics. (^∗^*p* < 0.05, ^∗∗^*p* < 0.01).

**FIGURE 8:**
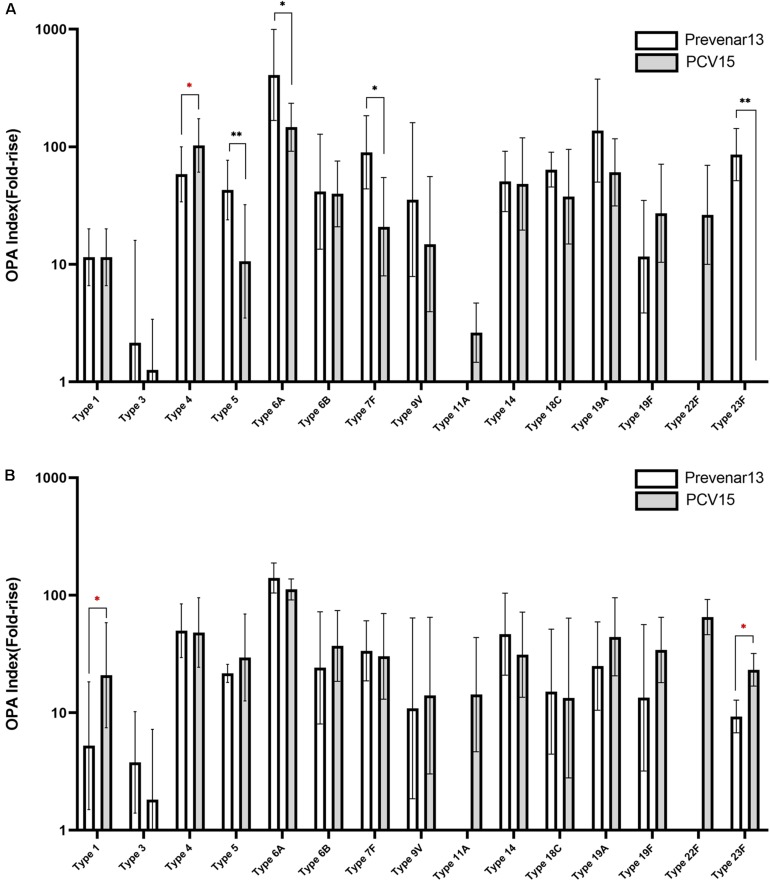
Immune responses against pneumococcal polysaccharides in mice measured by OPA. **(A)** Before the process optimization. **(B)** After the process optimization. Prevenar 13^®^ was used as a comparator vaccine and PCV15 indicates the polysaccharide-carrier protein conjugate produced in this study by Eubiologics. (^∗^*p* < 0.05, ^∗∗^*p* < 0.01).

A 15-valent conjugate vaccine prepared using CPS produced without process optimization was injected into rabbits, and sera were subsequently analyzed by ELISA and OPA. Significantly lower IgG levels for serotypes 1, 3, 5, 6A, 6B, 18C, 19A, and 23F were observed in the sera compared with those following administration of a comparator vaccine (Prevnar 13^®^), while IgG for serotypes 4, 7F, 9V, and 19F showed no significant differences (Figure 7A). When the CPS produced by the improved method was used in the conjugate vaccine, the concentration of IgG for most serotypes was equivalent to or higher than those observed after administration of Prevnar 13^®^, except for serotype 7, indicating that the polishing of the CPS production steps dramatically improved the quality of the vaccine and thus increased the immunogenicity of the vaccine (Figure 7B). In addition, IgG levels for serotypes 11A and 22F, which are not covered by Prevnar 13^®^, were similar to those for other serotypes covered by the 15-valent pneumococcal conjugate vaccine. OPA to measure the functional antibody levels in the sera also showed results similar to those of ELISA. Notably, serotypes 1, 5, 6B, 9V, 19A, and 23F in the method before improvement demonstrated lower opsonophagocytic killing activity than a comparator vaccine (Figure 8A); however, by improving the purification method and decreasing the CWPS, these serotypes showed higher or similar fold increases of OPA index (Figure 8B).

## Discussion

Several methods for purification of CPS from *S. pneumoniae* have been reported, and advances in technology have enabled production of CPS with high purity and yield (Suárez et al., 2001; Macha et al., 2014; Yuan et al., 2014; Zanardo et al., 2016; Morais et al., 2018). However, while these methods were optimized for specific serotypes of CPS such as type14 (Suárez et al., 2001; Zanardo et al., 2016), types 3, 6B, 14, 19F, and 23F (Macha et al., 2014), and types 1, 4, 5, 6A, 6B, 7F, 9V, 14, 18C, 19A, 19F, and 23F (Yuan et al., 2014), our method can be universally applied to all 15 serotypes (1, 3, 4, 5, 6A, 6B, 7F, 9V, 11A, 14, 18C, 19A, 19F, 22F, and 23F) contained in a newly developing 15-valent conjugate vaccine, although there is one determination step whether to use supernatant or precipitant depending on the charge of the target CPS after CTAB precipitation. Methods mentioned above do not use traditional CTAB precipitation which is our main separation step, and we combined this with other techniques including acid precipitation, ethanol precipitation, and HA chromatography and this ensured high purity of CPS and low CWPS contamination. Moreover, while most studies in the literature focus on reducing nucleic acid and protein impurities, we focused on the CWPS, which is one of the major contaminants in the process of CPS purification and remains a concern in the production of pneumococcal vaccines. Although CPS yield could have been compromised due to many steps which is the case in traditional methods (Morais et al., 2018), we substantially reduced CWPS contaminations in CPS. The method used in the development of Prevenar 13^®^, which is a comparator vaccine in our animal immunogenicity study, is based on CTAB precipitation as our technique (Hausdorff et al., 2010); however, the main difference is that it uses NaI to precipitate CTAB after CTAB precipitation and our method uses ethanol precipitation to specifically precipitate CPS.

## Conclusion

In conclusion, we developed our own pneumococcal CPS purification method by combining several traditional separation techniques such as ultrafiltration, CTAB precipitation, and chromatography; however, concerns about CWPS contamination remained, as high CWPS concentrations in CPS were observed by NMR spectroscopy. To solve this problem and reduce CWPS and other impurities, process optimization was carried out, which resulted in lower CWPS, leading to higher immunogenicity when used in a conjugate vaccine. By optimizing only the main purification processes, including extensive UF/DF and impurity precipitation, concentrations of CWPS were notably decreased. Moreover, in animal models, ELISA and OPA analyses revealed that the higher-quality CPS affected the immunogenicity of the multivalent pneumococcal conjugate vaccine in which CPS was used as an antigen. Our results reveal that the reduced levels of CWPS contamination of CPS by our optimized method significantly improved the quality of the CPS which can contribute to the development of new multivalent conjugate vaccines to prevent pneumococcal diseases.

## Data Availability Statement

The datasets generated for this study are available on request to the corresponding author.

## Ethics Statement

The animal study was reviewed and approved by the Institutional Animal Care and Use Committee of Chuncheon Bioindustry Foundation.

## Author Contributions

CL, SC, YB, and IL: conceptualization. CL, IL, and SP: data curation, supervision, and writing – review and editing. HC and CL: formal analysis. HC and RK: investigation. HC, MP, RK, and YW: methodology. HC: writing – original draft.

## Conflict of Interest

CL, HC, MP, RK, YW, SC, YB, and IL were employed by the company R&D Center, EuBiologics Co., Ltd. The remaining author declares that the research was conducted in the absence of any commercial or financial relationships that could be construed as a potential conflict of interest.
